# Plasticity in variation of xylem and phloem cell characteristics of Norway spruce under different local conditions

**DOI:** 10.3389/fpls.2015.00730

**Published:** 2015-09-10

**Authors:** Jožica Gričar, Peter Prislan, Martin de Luis, Vladimír Gryc, Jana Hacurová, Hanuš Vavrčík, Katarina Čufar

**Affiliations:** ^1^Department of Yield and Silviculture, Department of Forest Techniques and Economics, Slovenian Forestry InstituteLjubljana, Slovenia; ^2^Department Geografía, University of ZaragozaZaragoza, Spain; ^3^Faculty of Forestry and Wood Technology, Mendel University in BrnoBrno, Czech Republic; ^4^Department of Wood Science and Technology, Biotechnical Faculty, University of LjubljanaLjubljana, Slovenia

**Keywords:** cambium, growth/climate relation, *Picea abies*, tracheid, tracheidogram, cell differentiation, sieve cell, phloemogram

## Abstract

There is limited information on intra-annual plasticity of secondary tissues of tree species growing under different environmental conditions. To increase the knowledge about the plasticity of secondary growth, which allows trees to adapt to specific local climatic regimes, we examined climate–radial growth relationships of Norway spruce [*Picea abies* (L.) H. Karst.] from three contrasting locations in the temperate climatic zone by analyzing tree-ring widths for the period 1932–2010, and cell characteristics in xylem and phloem increments formed in the years 2009–2011. Variation in the structure of xylem and phloem increments clearly shows that plasticity in seasonal dynamics of cambial cell production and cell differentiation exists on xylem and phloem sides. Anatomical characteristics of xylem and phloem cells are predominantly site-specific characteristics, because they varied among sites but were fairly uniform among years in trees from the same site. Xylem and phloem tissues formed in the first part of the growing season seemed to be more stable in structure, indicating their priority over latewood and late phloem for tree performance. Long-term climate and radial growth analyses revealed that growth was in general less dependent on precipitation than on temperature; however, growth sensitivity to local conditions differed among the sites. Only partial dependence of radial growth of spruce on climatic factors on the selected sites confirms its strategy to adapt the structure of wood and phloem increments to function optimally in local conditions.

## Introduction

Norway spruce [*Picea abies* (L.) H. Karst.] is considered to have high adaptive potential and, despite the adverse effect of climate change on its growth, remains one of the most important European forest tree species (Skrøppa, 2003). Due to its major economic importance and vulnerability to climate change, several tree-ring studies have been carried out in recent decades (e.g., Bošel'a et al., 2014). Nevertheless, studies dealing with the plasticity of tree species, or climate reconstruction from tree rings, should not only focus on trees growing in relatively extreme sites (i.e., treeline or xeric habitats) but should also include various temperate habitats with moderate growing conditions, as has been recently stressed by several research groups (Carrer et al., 2012; Drew et al., 2013). Namely, most trees grow in forests that are not at the altitudinal or latitudinal limits of their distributions; consequently, large areas of the forested biomes of the world are ignored (Drew et al., 2013).

In dendrochronology, tree-ring widths are normally presented as time-series and are analyzed on an annual scale (Fritts, 1976). However, environmental information in trees is encoded in the inter- and intra-annual variability of tree-ring widths, including earlywood and latewood width, wood density, cellular wood structure, and chemical composition of cell walls (Eckstein, 2013). Cells are created in the wood formation process, which can be divided into five consecutive developmental stages: cell division, cell enlargement, cell wall thickening, lignification and programmed cell death (e.g., Rossi et al., 2014). The process depends on genetic signaling, availability of resources, temperature, tree water and nutrient status and the stage of ontogenetic development (Hölttä et al., 2010). Thus, to understand the mechanisms and the dynamics of wood formation in relation to climatic or physiological factors better, analyses on a shorter temporal scale are required (Rossi et al., 2014). Investigating the xylem anatomy level has already been demonstrated to be a promising approach in tree-ring studies (Fonti et al., 2010), while the environment–phloem relationship is still relatively unexplained. In general, the development of bark cells is very complex and not yet fully understood (Gričar et al., 2015). Although their main functions appear to be completely different, xylem and phloem are closely associated both spatially and functionally (Evert, 2006). There are two main long-distance pathways in trees, one related to the xylem conducting water and nutrients absorbed from the roots up to the leaves to sustain evapotranspiration and photosynthesis, and one related to the phloem delivering sugar solutions produced by photosynthesis that are necessary for cell respiration and growth to all the living tissues (Petit and Crivellaro, 2014). Carbon gain and whole-tree survival depend on the functioning of and interplay between these two vascular subsystems (e.g., Sevanto et al., 2014).

Research on the seasonal dynamics of phloem formation in various tree species (Prislan et al., 2013; Gričar et al., 2014b; Swidrak et al., 2014), as well as on the anatomy of phloem in relation to tree vitality (Gričar et al., 2014a), osmotic potential (Rosner et al., 2001) or variation along the stem (Petit and Crivellaro, 2014; Jyske and Hölttä, 2015), has noticeably increased in the last decade. Nevertheless, year-to-year variations in phloem cell characteristics in trees from different environments still remain relatively unknown. Secondary changes in older phloem tissue hinder its anatomical analysis in consecutive annual increments; only the youngest phloem increments are therefore appropriate for such observations (Gričar et al., 2015). As a result, sampling over several years needs to be performed to acquire a time series of phloem cell characteristics. In a recent study of Norway spruce, it was shown that the seasonal dynamics of phloem formation exhibited less plasticity than xylem formation in trees growing at the same location (Gričar et al., 2014b). However, differences in the phloem phenology among different sites suggest that phloem development is at least partly affected by local environmental conditions.

Intra- and inter-species variation in secondary tissues exists because of local adaptations and environmental conditions (Rowe and Speck, 2005). The capacity to change growth form may represent a significant advantage of plants when growing in changing environmental conditions (Agusti and Greb, 2013). There is limited information on intra-annual plasticity of variation of xylem and phloem cell characteristics of individual tree species under different environmental conditions. To increase the current knowledge about the plasticity of secondary growth that allows trees to adapt to specific environmental regimes (Rowe and Speck, 2005), we examined long-term radial growth of spruce from three contrasting locations in the temperate climatic zone by analyzing tree-ring widths for the period 1932–2010, and cell characteristics in xylem and phloem increments formed in the years 2009–2011. This joint approach of dendrochronological and quantitative-anatomical methods allowed to define climate-growth relationships from cell parameters and tree-ring widths more precisely. In order to obtain more information about the strategy of Norway spruce for functioning optimally in temperate local conditions, we addressed two main questions: (1) are there site-specific responses of wood and phloem anatomy to local conditions and (2) is year-to-year variability in environmental conditions more pronounced in the structure of xylem or phloem?

We analyzed the long-term tree ring-climate relationship, also separately for earlywood and latewood. The main novelty of this work is that it provides fundamental information on the variation in phloem anatomy over three consecutive years, in parallel with variations of xylem conduits in Norway spruce from three different sites with contrasting climate conditions.

## Materials and methods

### Study sites

**Figure 1:**
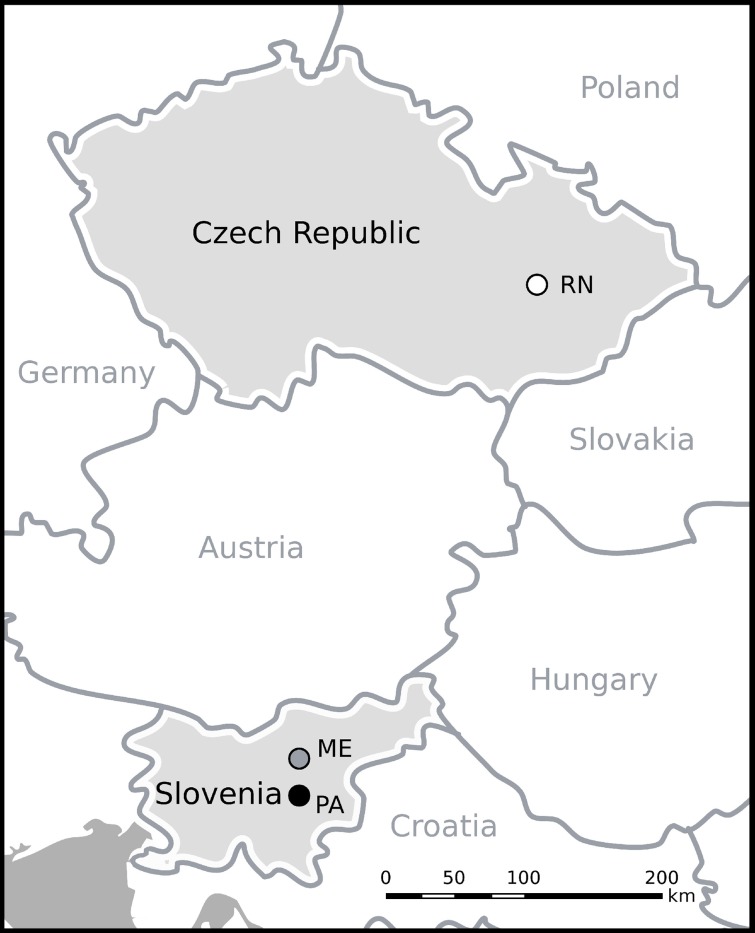
**Map with sampling locations Panška reka (PA) Menina planina (ME) and Rájec-Němčice (RN)**.

The study was carried out at three forest sites with different altitudes and latitudes: two in Slovenia and one in the Czech Republic. In Slovenia, sampling was performed at two uneven-aged mixed forest stands differing in altitude. The low elevation site, Panška reka (PA), is located near Ljubljana and is classified as *Hacquetio-fagetum typicum* forest type, where *Fagus sylvatica* L., *Acer pseudoplatanus* L. and *P. abies* (L.) H. Karst prevail (Table 1, Figure 1). The high elevation site, Menina planina (ME), is on a pre-Alpine Karst plateau in the Kamnik-Savinja Alps and is classified as *Abieti fagetum prealpinum typicum* forest type, with *F. sylvatica, P. abies*, and *Abies alba* Mill. being the dominant species. The site in the Czech Republic, Rájec-Němčice (RN), is located north of Němčice, ca. 400 km away from the Slovenian sites. The site is classified as *Abieto-Fagetum mesotrophicum* with *Oxalis acetosella* L. It is spruce monoculture (the first generation after mixed forest) (Fabiánek et al., 2009).

**Table 1:** **Location and characteristics of the study sites**.

**ID**	**Site**	**Country**	**Latitude**	**Longitude**	**Altitude (m a.s.l.)**	**Rock type**
PA	Panška reka	Slovenia	46°00′N	14°40′E	400	Dolomite
ME	Menina planina	Slovenia	46°16′N	14°48′E	1200	Limestone
RN	Rájec-Němčice	Czech Republic	49°29′N	16°43′E	650	Acid granodiorite

Climatic time series (minimum, maximum and mean monthly temperatures and monthly amount of precipitation) for the period 1901–2013 were obtained from the closest gridpoint of the CRU TS3.22 dataset (Harris and Jones, 2014). The general features of climatic regime at the three study sites are presented in Table 2.

**Table 2:** **Climate data: mean annual temperature (T), T range, mean annual amount of precipitation (P) and P range at PA, ME, and RN for the period 1901–2010**.

**Site**	**Mean annual T [°C]**	**T range [°C]**	**Mean annual amount of P [mm]**	**P range [mm]**
PA	10.3	8.9–12.2	1384	1091–1848
ME	7.4	4.2–13.0	1355	1125–1864
RN	8.1	3.8–12.4	661	364–977

### Tree selection and sample preparation for xylem and phloem analyses

We selected six dominant or co-dominant, healthy Norway spruce trees at each of the sites. The trees were 68 ± 8 (average ± standard deviation) years old at PA, 102 ± 31 at ME and 88 ± 4 at RN, with diameters at breast height (DBH) ranging from 30 to 40 cm (36 ± 5 cm at PA; 37 ± 12 cm at ME and 34 ± 2 cm at RN) and heights of around 25–32 m (30 ± 5 m at PA; 25 ± 1 m at ME and 32 ± 2 m at RN). For anatomical analysis of xylem and phloem increments, we used micro-cores that had been collected at the end of September/beginning of October in 2009, 2010, and 2011, when annual xylem and phloem increments were fully developed. They were taken at 1.1–1.7 m above ground using a Trephor tool (Rossi et al., 2006). In order to avoid wound effects, sampling locations were separated by 10 cm. Each micro-core contained phloem (non-collapsed and collapsed), cambium and at least two of the last-formed xylem rings. Immediately after removal from the trees, the samples were fixed in ethanol-formalin acetic acid solution (FAA). After 1 week, the samples were dehydrated in a graded series of ethanol, infiltrated with D-limonene and embedded in paraffin blocks (Rossi et al., 2006). Transverse sections of 8–12 μm thickness were cut with a rotary microtome, using low profile microtome blades. The sections were transferred to object glass and stained with a safranin (0.04%) and astra blue (0.15%) water mixture (van der Werf et al., 2007), dehydrated and embedded in Euparal. They were observed under a light microscope using transmission and polarized light modes. Histometrical analyses were performed with a digital camera and an image processing program ImageJ (Abramoff et al., 2004).

### Measurements of xylem and phloem cell characteristics and data processing

**Figure 2:**
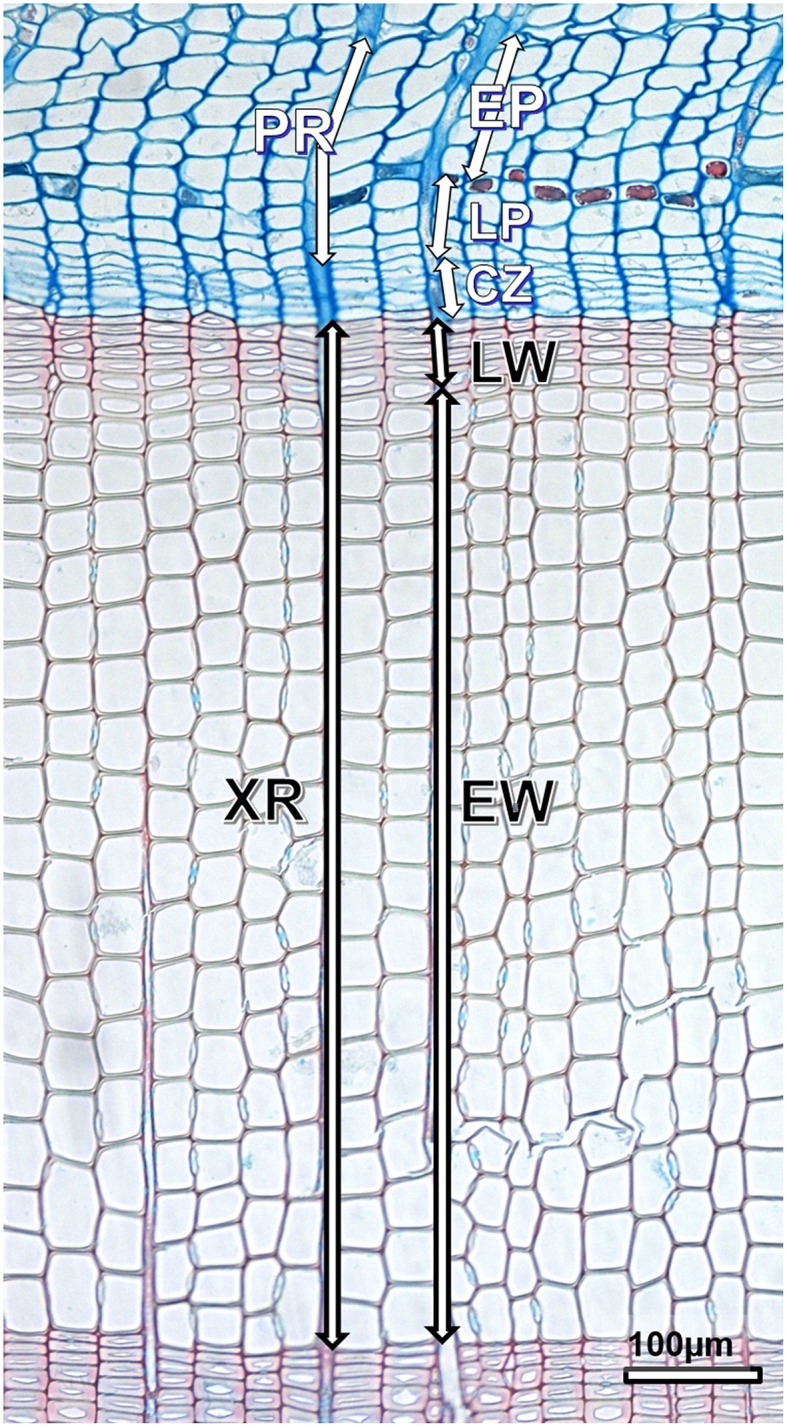
**Structure of xylem and phloem rings in ***Picea abies*****. Vertical bars denote widths of PR, phloem ring; EP, early phloem; LP, late phloem; CZ, dormant cambium; XR, xylem ring; EW, earlywood; and LW, latewood; scale bar = 100 μm.

In samples taken at the end of the vegetation periods of 2009–2011, xylem and phloem cell characteristics were measured immediately after the cessation of cambial activity and cell differentiation processes (Figure 2). This was important because, early phloem sieve cells already started to collapse in the autumn of the current growing season, which we tried to avoid. Vaganov (1990) used the term “tracheidogram” for plots showing the variation of tracheid parameters in the growing period of a conifer. By analogy, we called the plots of variations in phloem cell size (i.e., sieve cells and axial parenchyma cells) in the growing season “phloemograms.”

Measurements were performed on transverse sections along three radial cell rows per growth ring. Only rows in which cells were cut approximately in the middle of their longitudinal length (i.e., cells with maximum tangential and radial diameter) were chosen. In the phloem, we analyzed cells that were not crushed. Mean values of the listed anatomical variables for earlywood/latewood and early phloem/late phloem separately were calculated for each cell row. Since the number of cells in radial rows of the xylem or phloem annual rings was different, it was necessary to standardize the sample size per year in order to compare them among trees, sites and years. We therefore used the “relative position” of each cell within a radial row of an annual ring instead of an absolute value.

In xylem increments, we analyzed the following variables: ring width expressed in number of cells (mean of three radial files/tree), number of earlywood and latewood cells (mean of three radial files/trees), radial diameter of tracheids (mean of three radial files/tree), lumen diameter of tracheids (mean of three radial files/tree), and double cell wall thickness of tracheids (mean of three radial files/tree) for each cell in a radial row. Finally, the mean values of initial earlywood (i.e., first tangential row of cells at the growth ring boundary) and terminal latewood tracheids (i.e., last tangential row of cells adjacent to the cambium) were calculated. Data for each anatomical variable per file were transformed into a tracheidogram. Latewood tracheids were defined according to Denne's (1988) first interpretation of Mork's (1928) definition; i.e., when the width of the cell lumen was smaller than twice the double cell wall thickness.

In phloem increments, we assessed five anatomical variables: ring width expressed in number of cells (mean of three radial files/tree), number of early and late phloem cells (mean of three radial files/trees), radial diameter (mean of three radial files/tree) and number of sieve cells (mean of three radial files/tree) for each cell in a radial row. Finally, the mean values of initial early phloem (i.e., first tangential row of cells at the phloem growth ring boundary) and terminal late phloem sieve cells (i.e., last tangential row of cells adjacent to the cambium) were calculated. The transition from early to late phloem was identified by the appearance of the first axial parenchyma cells separating the two tissues, which is typical of Pinaceae.

### Tree-rings and chronology computation

At all sites, we took samples of wood (cores or discs) for dendroclimatological analysis from 13–16 dominant or co-dominant Norway spruce trees growing near to each other and having approximately the same diameters, heights and ages as the trees used for analysis of xylem and phloem anatomy. The samples of wood taken from trees after the end of 2010 (PA, ME) and 2011 (RN) growing seasons, were polished and the tree-ring widths, as well as visually separated earlywood and latewood widths, were measured to the nearest 0.01 mm. TSAP Win or WinDENDRO programs were used for data acquisition and cross-dating.

The tree-ring series were assembled into local chronologies using the ARSTAN programme (Holmes, 1994). The individual raw tree-ring series were standardized in a two-step procedure. First, the long-term trend was removed by fitting a negative exponential function or a regression line to each tree-ring series. Second, more flexible detrending was achieved by a cubic smoothing spline with a 50% frequency response of 60 years, in order to reduce non-climatic variance further. Thereafter, bi-weight robust estimation of the mean was applied to construct local standardized chronologies (Cook and Peters, 1997).

### Anatomical variables and climate-growth relationships

Pearson's Product Moment Correlation Coefficient was used to measure the strength of association between xylem and phloem ring widths, earlywood, and latewood widths, early phloem and late phloem widths, and number of cells and measured width of xylem ring at PA, ME, and RN. The differences among years and sites in the listed xylem and phloem anatomical variables were determined using individual One-Way repeated measures ANOVA, in which the site was the treatment factor and year of growth was the repeated measure. The normality of distribution and homogeneity of variance were verified using the Shapiro-Wilk *W*-test and Levene's test, respectively (Quinn and Keough, 2002).

Climate–growth relationships were calculated through correlation function analysis using the program DendroClim2002 (Biondi and Waikul, 2004), whereby the standardized chronologies of the tree-ring chronology was the dependent variable and the regressors were the monthly minimum, maximum and mean temperatures and the monthly sums of precipitation for each year from the previous September to the current October. Analyses were based on the common 1931–2010 period, in which an elevated Expressed Population Signal (EPS > 0.85) was observed in all three chronologies. Relationships between climatic conditions and xylem and phloem anatomical variables for the period 2009–2011 were also calculated. For each site and year, mean values of each analyzed variable were compared with the monthly minimum, maximum and mean temperatures and the monthly sums of precipitation from previous September to current October using Pearson correlation. Procedure was the same as for dendrocronological analyses, however here we used 3 sites together and 3 years (9 values).

## Results

### Xylem and phloem cell characteristics

In general, we found statistically significant differences in xylem and phloem anatomical variables among the selected sites but not among the years (Table 3). Only the average double cell wall thickness of earlywood tracheids and of initial earlywood tracheids also statistically differed among the years. On the other hand, no significant differences were found in the number of earlywood cells, the radial (lumen) dimension of initial earlywood tracheids and radial dimension of terminal latewood and late phloem cells. In the case of the number of latewood and late phloem cells, as well as the radial dimensions of initial early phloem cells, we detected differences only between some sites and some years, showing an inconsistent site effect and thus high variation in the values of these anatomical parameters. Correlation between number of cells and measured width of xylem ring was 0.98, between xylem and phloem ring widths 0.98, between earlywood and latewood widths 0.23, and between early phloem and late phloem widths 0.41.

**Table 3:** **Summary of one-way repeated measures ANOVA of anatomical variables in which “site” was the treatment factor and “year of growth” was the repeated measure**.

**Anatomical variable**	**Site**		**Year**		**Site** ^*^ **Year**	
	***F***	***p***	***F***	***p***	***F***	***p***
Number of xylem cells	**4.50**	**0.028**	2.95	0.105	**3.90**	**0.042**
Number of earlywood cells	2.53	0.111	0.50	0.488	0.32	0.730
Number of latewood cells	**9.67**	**0.002**	1.62	0.222	**6.50**	**0.009**
Proportion of earlywood cells in xylem ring	**15.84**	**0.000**	0.31	0.586	3.42	0.058
Average double cell wall thickness of earlywood tracheids	**16.14**	**0.000**	**11.40**	**0.004**	1.26	0.312
Average double cell wall thickness of latewood tracheids	**13.17**	**0.000**	1.10	0.309	0.92	0.421
Average lumen dimension of earlywood tracheids	**4.38**	**0.030**	2.56	0.129	0.97	0.400
Average lumen dimension of latewood tracheids	3.28	0.064	1.09	0.313	1.77	0.203
Double cell wall thickness of initial earlywood tracheid	**11.77**	**0.001**	**7.52**	**0.014**	1.92	0.179
Double cell wall thickness of terminal latewood tracheid	**8.02**	**0.004**	0.62	0.444	0.61	0.555
Radial lumen dimension of initial earlywood tracheid	0.77	0.480	2.38	0.143	0.77	0.478
Radial lumen dimension of terminal latewood tracheid	**7.52**	**0.005**	1.35	0.262	1.22	0.321
Number of phloem cells	**24.73**	**0.000**	0.16	0.697	2.37	0.127
Number of early phloem cells	**19.10**	**0.000**	0.88	0.364	1.69	0.217
Number of late phloem cells	**23.84**	**0.000**	0.00	1.000	**4.75**	**0.025**
Proportion of early phloem cells in phloem ring	**30.37**	**0.000**	0.03	0.855	**9.15**	**0.003**
Average radial dimensions of early phloem sieve cells	**20.50**	**0.000**	0.46	0.506	1.42	0.273
Average radial dimensions of late phloem sieve cells	**17.22**	**0.000**	0.05	0.827	0.91	0.423
Radial dimension of initial early phloem sieve cell	**8.60**	**0.003**	0.06	0.814	4.61	0.027
Radial dimension of terminal late phloem sieve cell	0.73	0.497	0.19	0.666	0.06	0.947
Average radial dimension of axial parenchyma cells	**3.95**	**0.042**	1.66	0.218	0.20	0.824

*Statistically significant values are in bold*.

**Figure 3:**
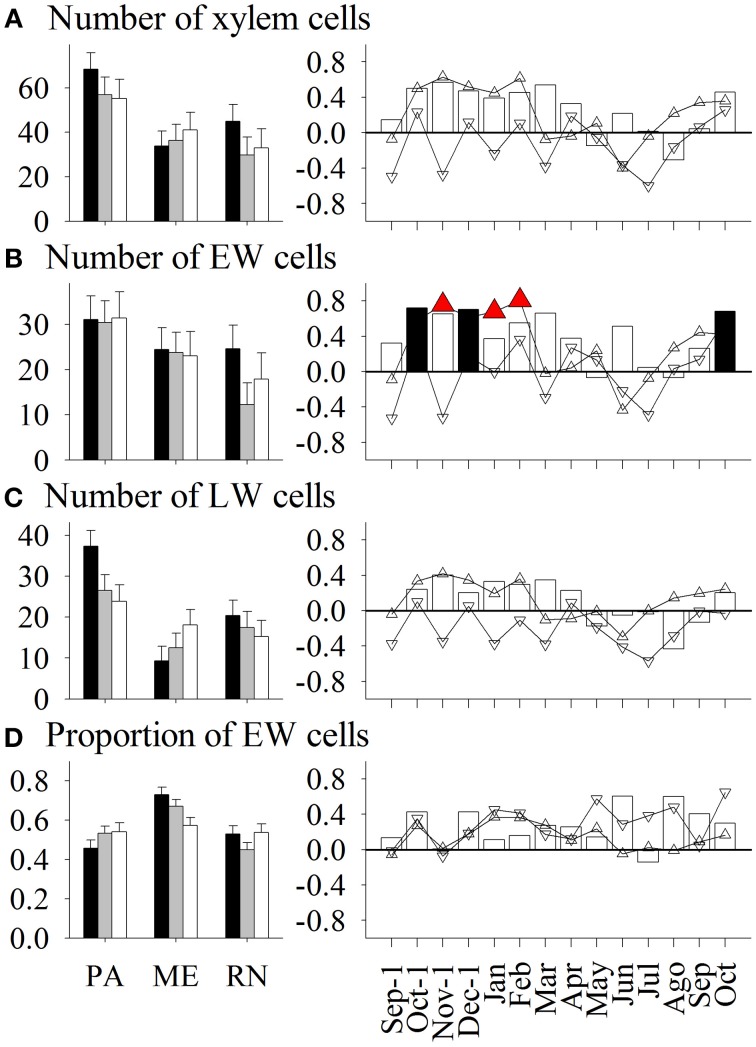
**Left figure:** Mean values of number of **(A)** xylem cells, **(B)** earlywood (EW) cells, **(C)** latewood (LW) cells, and **(D)** proportion of earlywood cells in *Picea abies* at Panska reka (PA), Menina planina (ME) and Rájec-Němčice (RN) in 2009 (black columns), 2010 (grey columns) and 2011 (white columns). Error bars correspond to 95% confidence intervals. **Right figure:** Correlation coefficients calculated between mean values and climatic conditions in this period. Significance at a 95% level indicates the following symbols: red triangles up for maximum temperature and black columns for precipitation.

**Figure 4:**
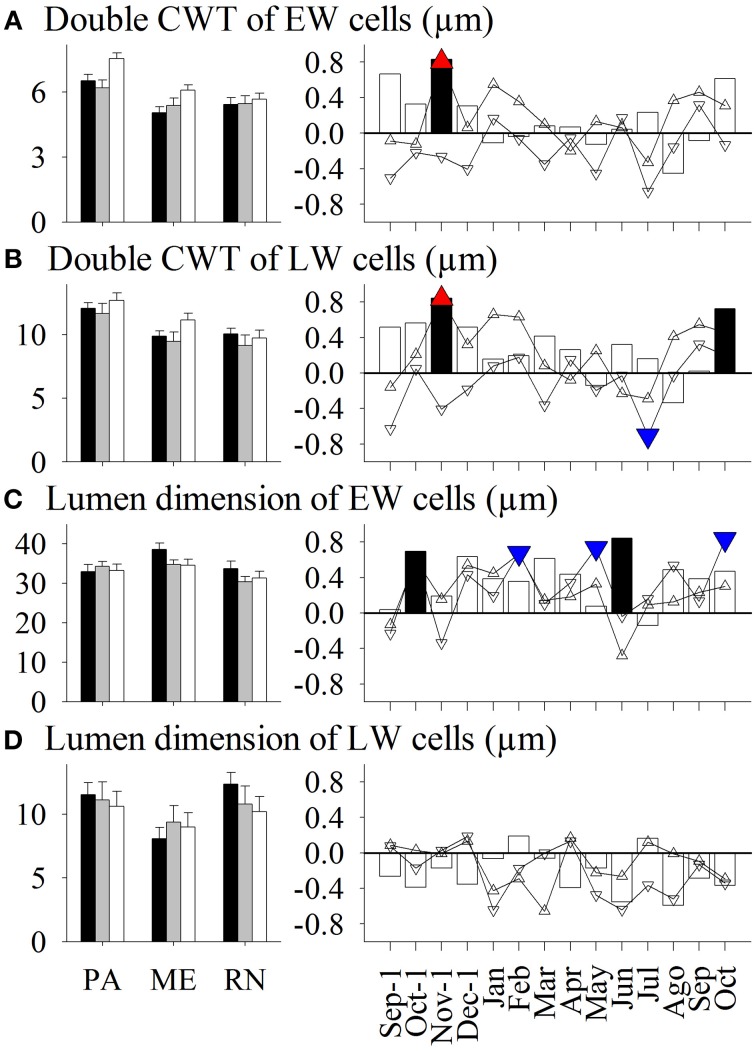
**Left figure:** Mean values of double cell wall thickness (CWT) of **(A)** earlywood (EW) cells and **(B)** latewood (LW) cells, and lumen dimension of **(C)** earlywood cells and **(D)** latewood cells in *Picea abies* at Panška reka (PA), Menina planina (ME) and Rájec-Němčice (RN) in 2009 (black columns), 2010 (gray columns), and 2011 (white columns). **Right figure:** Correlation coefficients calculated between mean values and climatic conditions in this period. Significance at a 95% level indicates the following symbols: red triangles up for maximum temperature, blue triangles down for minimum temperature and black columns for precipitation.

**Figure 5:**
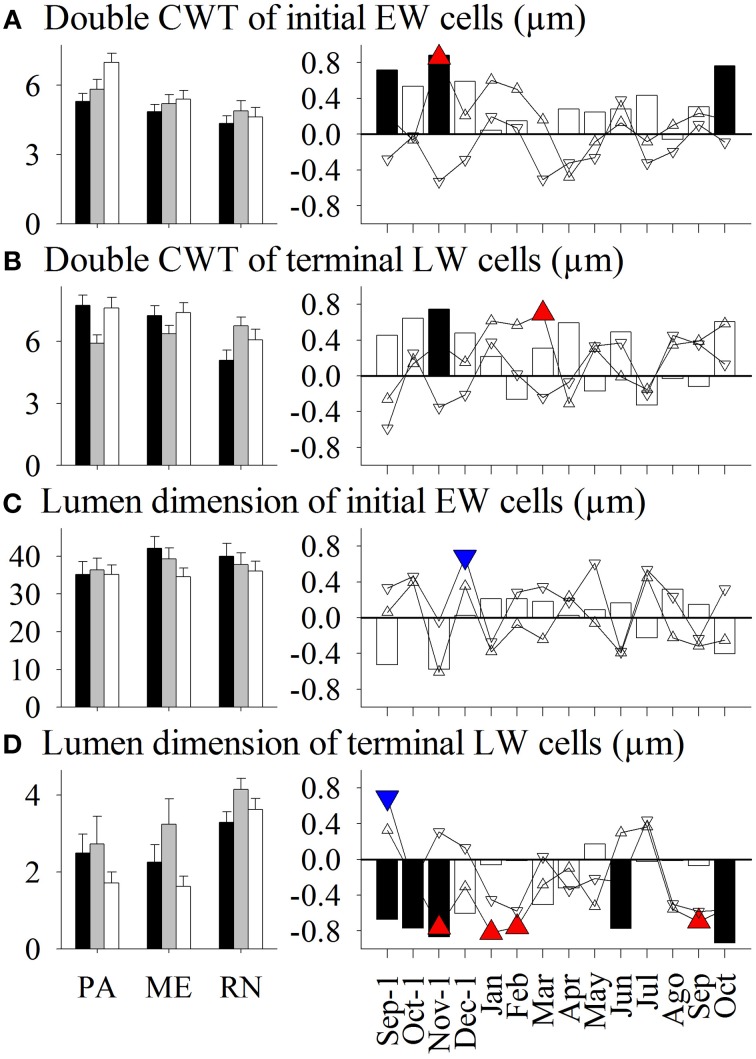
**Left figure:** Mean values of double cell wall thickness (CWT) of **(A)** initial earlywood (EW) cells and **(B)** terminal latewood (LW) cells, and lumen dimension of **(C)** initial earlywood cells and **(D)** terminal latewood cells in *Picea abies* at Panška reka (PA), Menina planina (ME) and Rájec-Němčice (RN) in 2009 (black columns), 2010 (gray columns), and 2011 (white columns). **Right figure:** Correlation coefficients calculated between mean values and climatic conditions in this period. Significance at a 95% level indicates the following symbols: red triangles up for maximum temperature, blue triangles down for minimum temperature and black columns for precipitation.

**Figure 6:**
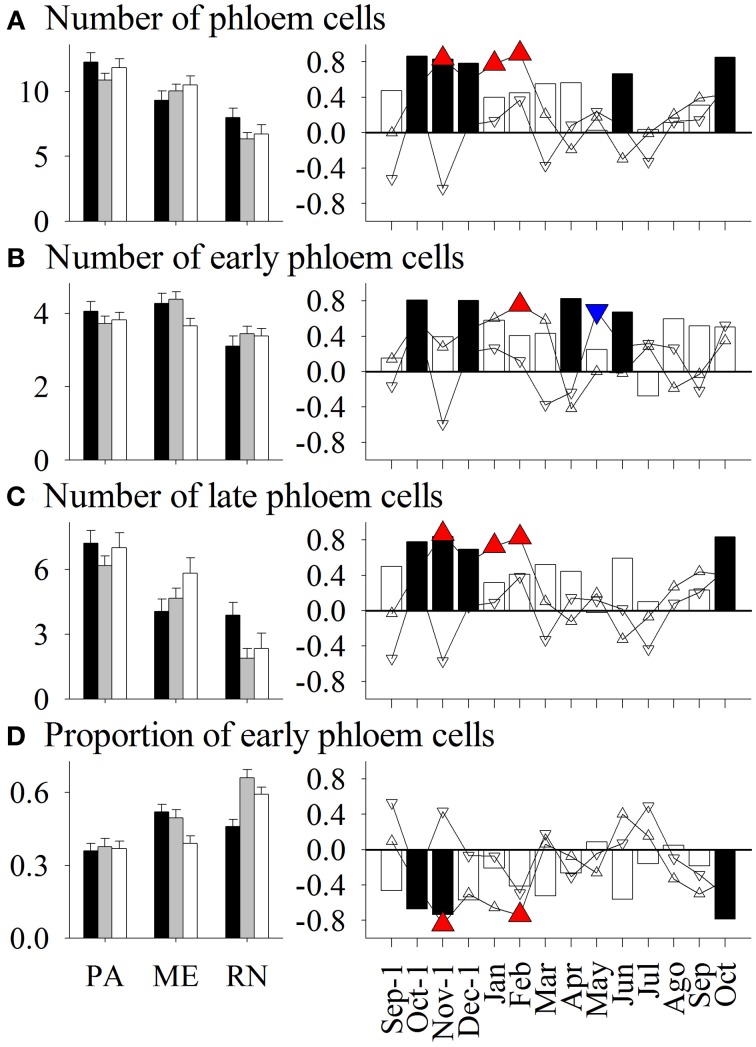
**Left figure:** Mean values of number of **(A)** phloem cells, **(B)** early phloem cells, **(C)** late phloem cells, and **(D)** proportion of early phloem cells in *Picea abies* at Panška reka (PA), Menina planina (ME), and Rájec-Němčice (RN) in 2009 (black columns), 2010 (gray columns), and 2011 (white columns). Error bars correspond to 95% confidence intervals. EW, earlywood; LW, latewood. **Right figure:** Correlation coefficients calculated between mean values and climatic conditions in this period. Significance at a 95% level indicates the following symbols: red triangles up for maximum temperature, blue triangles down for minimum temperature and black columns for precipitation.

**Figure 7:**
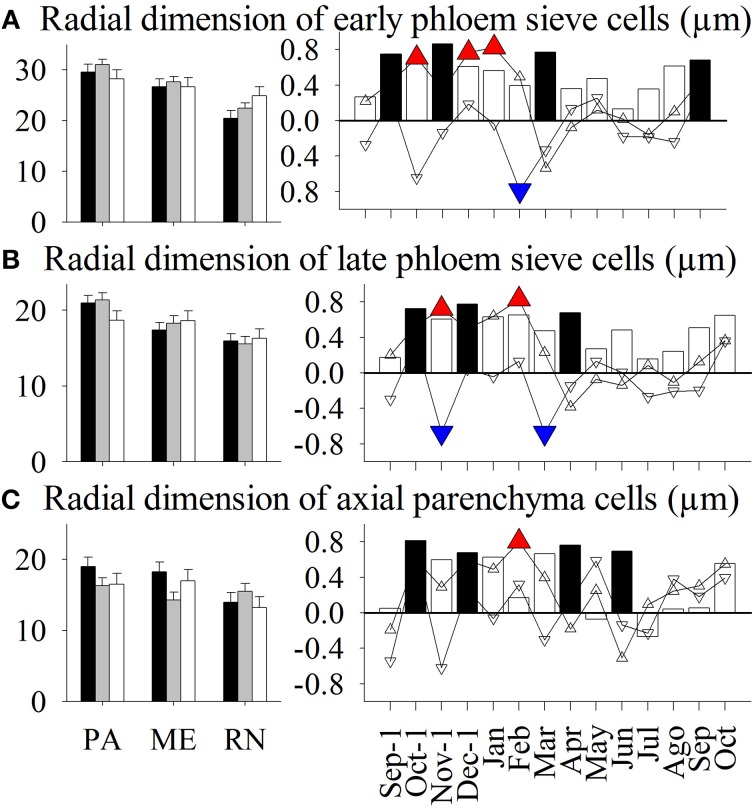
**Left figure:** Mean values of radial dimension of **(A)** early phloem sieve cells, **(B)** late phloem sieve cells, and **(C)** axial parenchyma cells in *Picea abies* at Panška reka (PA), Menina planina (ME), and Rájec-Němčice (RN) in 2009 (black columns), 2010 (gray columns), and 2011 (white columns). **Right figure:** Correlation coefficients calculated between mean values and climatic conditions in this period. Significance at a 95% level indicates the following symbols: red triangles up for maximum temperature, blue triangles down for minimum temperature and black columns for precipitation.

**Figure 8:**
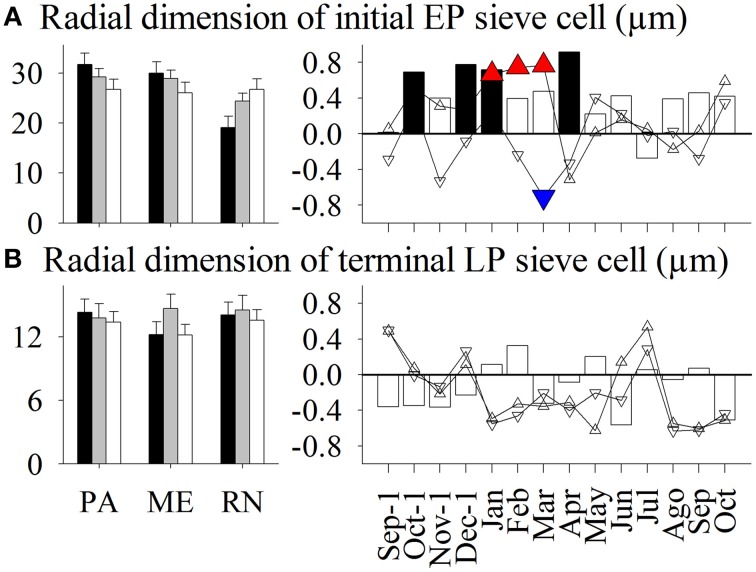
**Left figure:** Mean values of radial dimension of **(A)** initial early phloem sieve (EP) cells and **(B)** terminal late phloem (LP) sieve cells in *Picea abies* at Panška reka (PA), Menina planina (ME), and Rájec-Němčice (RN) in 2009 (black columns), 2010 (gray columns), and 2011 (white columns). **Right figure:** Correlation coefficients calculated between mean values and climatic conditions in this period. Significance at a 95% level indicates the following symbols: red triangles up for maximum temperature, blue triangles down for minimum temperature and black columns for precipitation.

Spruce at PA thus had on average the widest xylem and phloem increments, latewood and late phloem, as well as the thickest walls of earlywood and latewood tracheids (Figures 3–**8**). The radial dimensions of phloem sieve cells were proportionally related to phloem width (**Figures 7**, **8**). Xylem annual increments were narrowest at ME, while phloem increments were narrowest at RN. Trees at ME had the highest proportion of earlywood, widest early phloem and widest average radial (lumen) dimension of earlywood tracheids. At RN, only the lumen dimensions of latewood cells were significantly wider than at the other two sites. Phloem rings at ME and RN were about 20 and 40%, respectively, narrower than at PA (**Figure 6**). Furthermore, spruce at RN had about 16 and 12%, respectively, narrower early phloem and late phloem sieve cells than at ME and PA.

Early phloem sieve cells were on average 30% wider than late phloem cells at all three sites. The lumen dimensions of early phloem sieve cells were 12–30% smaller than those of earlywood tracheids. In the increments formed in the second part of the growing season, it was just the opposite: lumen dimensions were 30–50% smaller in latewood than in late phloem. A tangential band of axial parenchyma that normally separates early and late phloem was discontinuous in narrower phloem rings, composed of fewer than six cell layers, which were detected only in spruce at RN in 2010 and 2011. In contrast, an additional discontinuous tangential band of axial parenchyma was detected in the late phloem of increments wider than 12 cell layers, as observed at PA.

Only some variations in anatomical variables in xylem and phloem were explained by seasonal climatic data (Figures 3–8). Thus, the number of earlywood cells was positively influenced by precipitation in the previous October and December, and maximum temperature in the period November–February (Figure 3). The wall thickness of xylem cells was mainly influenced by maximum temperature and precipitation of the previous November, and in the case of latewood cells by minimum temperature in July and precipitation in October. The lumen dimension of earlywood tracheids was affected by precipitation in the previous October and current June, and by minimum temperature in February and May. Precipitation in the previous autumn and current June, and maximum temperature in winter negatively influenced lumen dimensions of terminal latewood tracheids (Figures 4, 5). The number of (late) phloem cells as well as their radial dimensions were mainly affected by precipitation in late autumn and maximum temperature in winter (Figures 6–8). The number of early phloem cells and dimension of all phloem cells were also positively influenced by precipitation in April. In addition, radial dimension of initial early phloem sieve cells was predominantly determined by maximum temperature in the period January–March.

**Figure 9:**
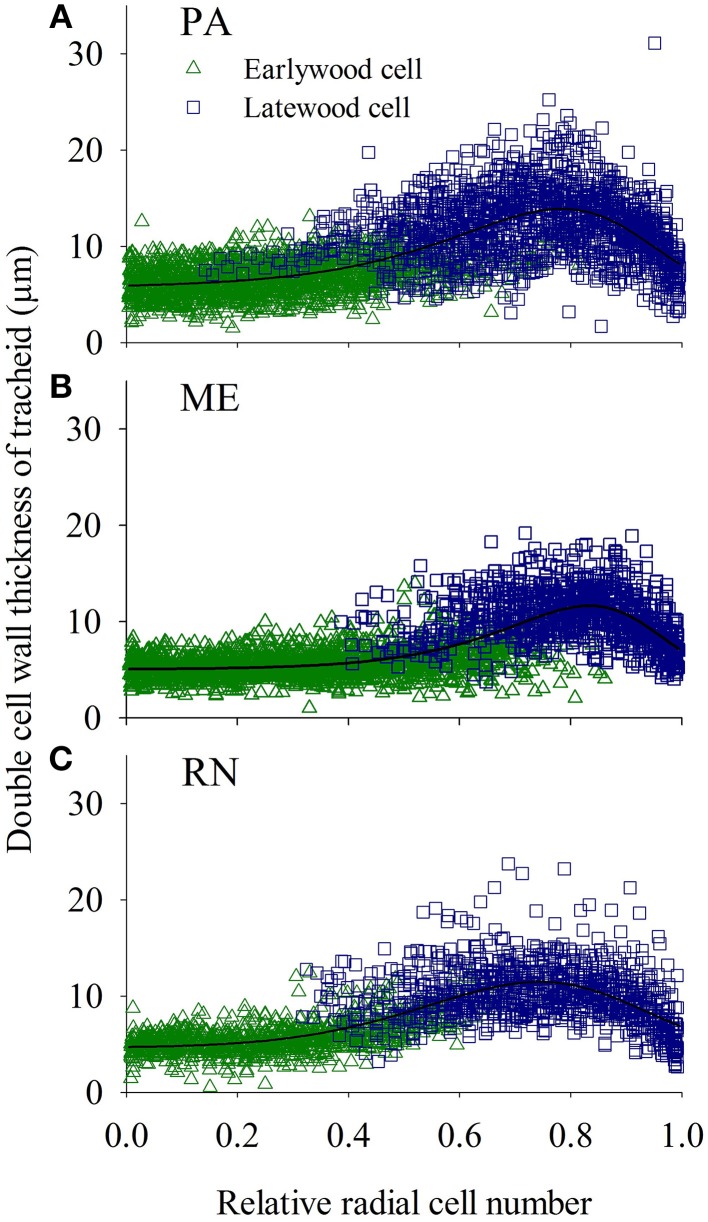
**Tracheidograms presenting double cell wall thickness of tracheids of spruce at PA (A), ME (B) and RN (C) for the period 2009–2011**. “Weibull function” was used for double cell wall thickness and “Logistic function” for lumen dimension as a reference line.

**Figure 10:**
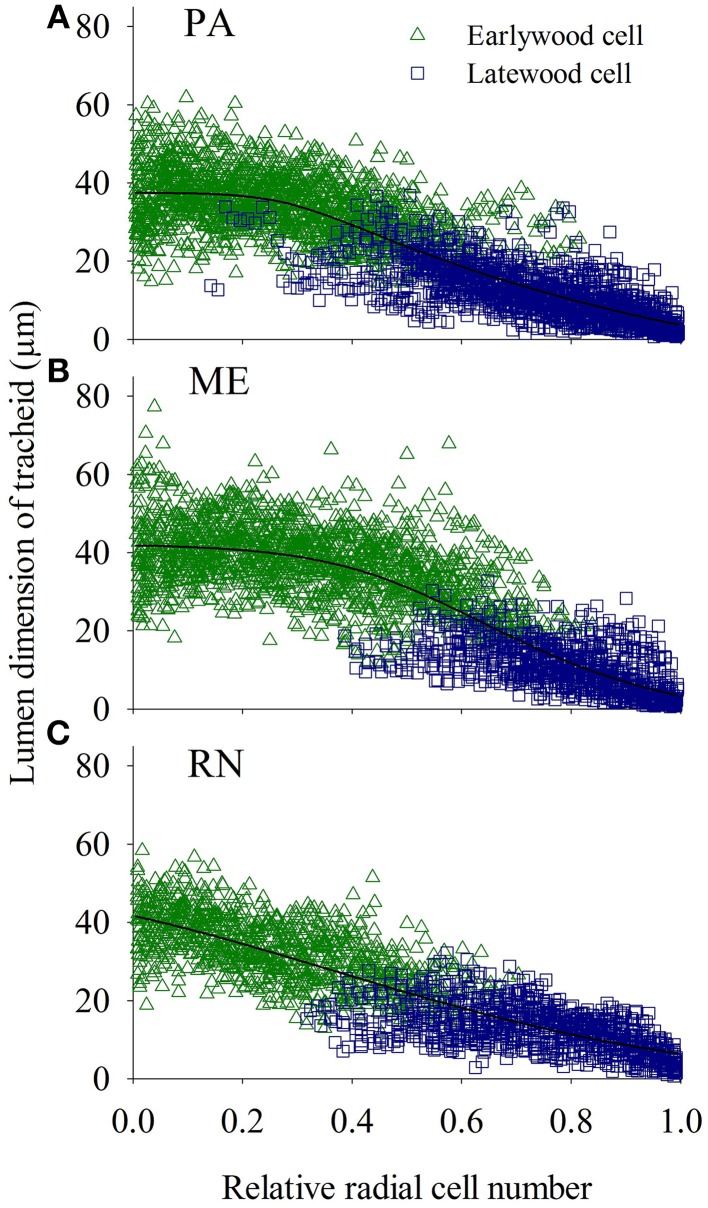
**Tracheidograms presenting lumen dimension of tracheids of spruce at PA (A), ME (B) and RN (C) for the period 2009–2011**. “Weibull function” was used for double cell wall thickness and “Logistic function” for lumen dimension as a reference line.

**Figure 11:**
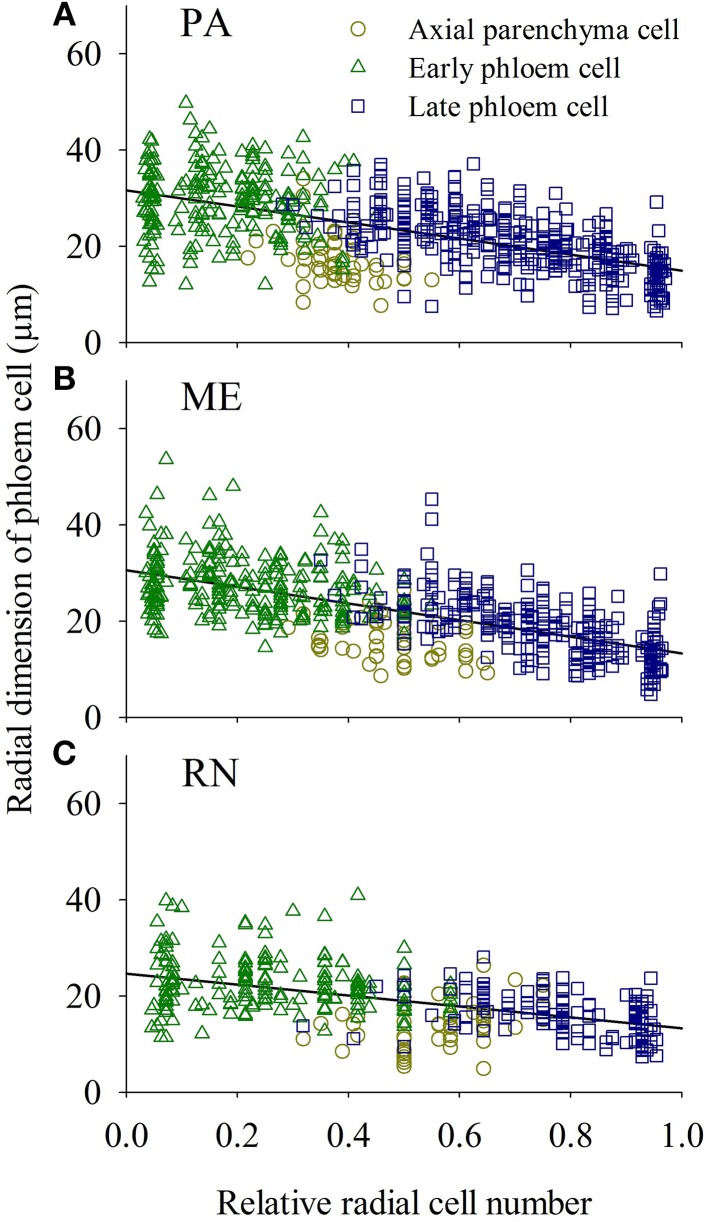
**Phloemograms presenting radial dimensions of phloem cells of spruce at PA (A), ME (B) and RN (C) for the period 2009–2011**. “Linear function” was used as a reference line.

Cell wall thickness of the tracheids along the radial files slowly increased and reached maximum values in latewood cells at a 0.7–0.9 relative position within a radial row and afterwards again decreased (Figure 9). Radial dimensions of xylem and phloem cells showed a different trend; cells formed at the beginning of the growing season grew larger than those created later in the growing season (Figures 10, 11).

### Long-term xylem-climate relations

**Figure 12:**
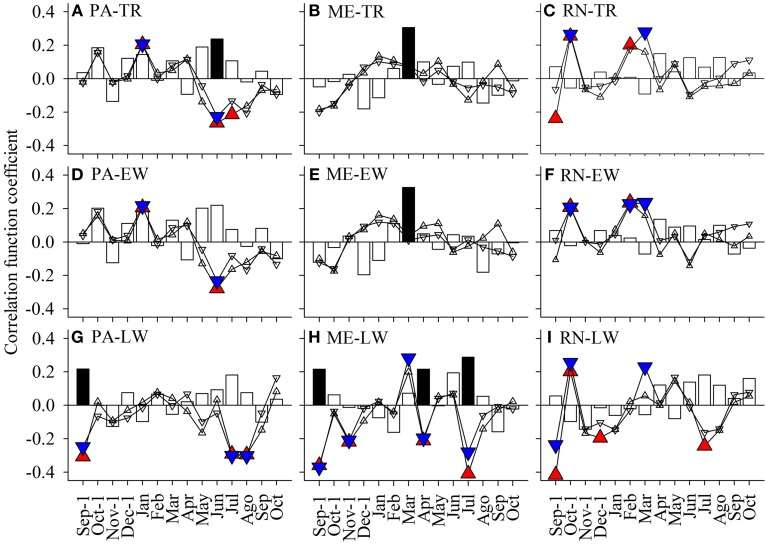
**Bootstrap correlation values calculated between monthly minimum (triangles down) and maximum temperature (triangles up) and precipitation (bars) from the previous September to the current October and residual spruce chronologies of widths of tree-ring (A–C), earlywood (D–F), and latewood (G–I) of ***Picea abies*** from PA, ME, and RN for the period 1933–2010**. Significance at a 95% level indicates the following symbols: red triangles up for maximum temperature, blue triangles down for minimum temperature and black columns for precipitation.

The main descriptive statistics of the local tree-ring, earlywood and latewood chronologies of spruce at PA are presented in Table 4. For spruce at PA, summer (June, July) temperature (where both minimum and maximum temperature had negative affect) proved to be the most important climatic factor explaining year-to-year variations in the widths of xylem increments (Figures 12A,D,G). In addition, June temperature negatively affected earlywood width, whereas July and August temperature negatively affected latewood width. Furthermore, January temperature positively influenced tree-ring and earlywood widths, while the previous September's temperature and precipitation influenced latewood widths. At ME, tree-ring and earlywood widths were positively related to March precipitation (Figures 12B,E). In the case of latewood, widths were influenced by the climatic conditions of the previous autumn (September and November), of early spring (March and April) and of July (Figure 12H). The temperature of the previous September and October was important for explaining year-to-year variations in the widths of xylem increments at RN (Figures 12C,F,I). Furthermore, February maximum and March minimum temperature was significant for tree-ring and earlywood widths, whereas July maximum temperature negatively affected latewood widths at this site.

**Table 4:** **Descriptive statistics for detrended standard chronologies of tree-ring, earlywood and latewood widths of ***Picea abies*** from PA, ME, and RN**.

	**Standardized chronologies**		
	**PA**	**ME**	**RN**
Time span	1932–2010	1861–2010	1916–2010
Length	79	150	96
Average length of included series	53	84	78
**TREE-RING**			
Mean ring width	0.995	0.994	0.980
Standard deviation	0.142	0.132	0.181
Skewness	0.633	−0.094	−0.471
Kurtosis	2.212	−0.290	1.243
Mean sensitivity	0.142	0.136	0.168
Autocorrelation order 1	0.114	0.220	0.344
Mean correlation between trees	0.327	0.381	0.426
Signal-to-noise ratio	6.809	6.164	11.89
Agreement with population chronology	0.872	0.860	0.922
**EARLYWOOD**			
Mean earlywood width	0.994	0.993	0.975
Standard deviation	0.152	0.142	0.201
Skewness	0.743	−0.007	−0.364
Kurtosis	2.189	−0.193	0.904
Mean sensitivity	0.159	0.147	0.205
Autocorrelation order 1	0.056	0.175	0.259
Mean correlation between trees	0.266	0.375	0.398
Signal-to-noise ratio	4.719	5.989	10.588
Agreement with population chronology	0.825	0.857	0.914
**LATEWOOD**			
Mean latewood width	0.979	0.983	0.976
SD	0.172	0.200	0.177
Skewness	−0.027	−0.058	−0.260
Kurtosis	−0.014	−0.258	0.871
Mean sensitivity	0.181	0.223	0.177
Autocorrelation order 1	0.101	0.420	0.226
Mean correlation between trees	0.257	0.340	0.263
Signal-to-noise ratio	4.491	4.628	5.719
Agreement with population chronology	0.818	0.822	0.851

## Discussion

### Xylem cell characteristics

To cope with different weather conditions, trees have evolved phenological, morphological, and physiological adaptations, which often arise from specific patterns of cambial activity and cell differentiation and are responsible for changes in xylem-hydraulic conductivity and vulnerability to cavitation (Eilmann and Rigling, 2012). We showed that xylem anatomical parameters generally varied among the sites but not among the years, indicating a fairly stable structure at a given location with almost no year-to-year variation. Spruce at the lower elevation PA had on average the widest xylem increments and narrowest at the Czech site RN. These differences could not be attributed to climatic factors as seen from climate-cellular relationship in xylem and phloem for the period 2009–2011. This confirms our previous speculations that local adaptation may also play a decisive part in the strategy of spruce for adapting the structure of wood and phloem increments to function optimally in local conditions (Gričar et al., 2014b). Further, earlywood proportion was the highest in spruce at the Alpine site ME. The number of earlywood cells was positively influenced by climatic conditions in late autumn and winter. Because photosynthesis is possible in conifers in winter (Guehl, 1985), high temperature during the winter period, in combination with a sufficient water supply, could promote growth and improve carbon storage in the following year (Battipaglia et al., 2009; Lebourgeois et al., 2010). In addition, high winter or early spring temperatures may trigger cambial reactivation (e.g., Begum et al., 2013).

The average lumen dimension of earlywood tracheids was largest at ME, whereas the lumen diameter of initial tracheids was comparable at all sites. The lumen dimension of earlywood tracheids was affected by precipitation in the previous October and current June, and by minimum temperature in February and May. Tracheid enlargement is irreversible and drought sensitive processes, and is largely determined by the ability of the cell to generate and maintain positive turgor (Hölttä et al., 2010). Thus, the radial diameter of tracheids is directly affected by changes in water availability during the period of cell enlargement (e.g., Abe et al., 2003), but may also be controlled by temperature months before the tissue is formed (Eckstein, 2013). In addition, the cell size can be influenced by numerous factors besides limited water availability: period of cell enlargement, the limitation of assimilates and a reduced synthesis of growth regulators, which are essential for cell enlargement (Eilmann et al., 2006). A higher earlywood proportion indicates the effective water use of trees (Domec and Gartner, 2002). Although the relative proportion of earlywood decreased with tree-ring width, the absolute number of earlywood cells was higher in wider rings (even if statistically not significant), which would explain the smaller values for the average lumen diameter of earlywood cells in spruce at PA, but similar values for initial tracheids. The more or less constant width of earlywood suggests its priority for the tree; a conduction function over a mechanical one. As stressed by Anfodillo et al. (2013), variation of wood traits is dependent on a tree's size. The conduit diameter is thus strictly related to tree height; in a basipetal direction, it is gradually wider. The distance from the stem apex is therefore crucial for comparison of the conduit diameters among different trees and sites. The trees selected for our study were already mature, with comparable heights, so the hydraulic parameters were probably not affected by changes in tracheid size.

Compared to earlywood width, latewood width, as well as the thickness of cell walls in latewood, seemed to be more variable, whereas the average lumen dimension of latewood tracheids was stable. Only lumen dimension of terminal latewood tracheids was affected by climatic conditions. A decreased latewood proportion in narrower rings would negatively affect the density and mechanical properties of wood but perhaps the need for additional strength provided by latewood becomes less crucial as the stem increases in diameter (Rao et al., 1997). Similarly, Park and Spiecker (2005) observed that cell parameters in earlywood changed very little from site to site, whereas those in latewood were more influenced by site conditions. Trees from a warm-dry site also had more latewood cells and substantially thicker cell walls, whereas those from a cool-humid site had larger earlywood cells. Authors have ascribed these differences to the hydraulic adaptation mechanisms of trees to given site conditions (Park and Spiecker, 2005).

The wall thickness of the tracheids in the annual xylem ring followed a Weibull function pattern, indicating that terminal latewood tracheids did not have the thickest cell walls. Furthermore, the cell walls of the terminal latewood tracheids were thinnest in spruce from the lower site PA, where cambial cell production ceased later than at the other two sites (Gričar et al., 2014b). Consequently, development of the last formed cells also finished later (Gričar et al., 2005). The end of final cell maturation was thus basically proportionally related to the date of the cessation of cambial activity, and inversely related to the elevation. It has been previously reported for treeline Norway spruce that maximum wood density (and the thickest cell walls) might not be located exactly in the terminal cell row of a tree ring, because lignification of the secondary cell wall in terminal latewood tracheids is also influenced by the climatic conditions, particularly in September (Gindl et al., 2000). It could be speculated that earlier cessation of cambial cell production would lead to thicker cell walls of terminal latewood tracheids because their development would occur earlier in autumn, when the temperature is higher. We found that the wall thickness of latewood cells were mainly influenced by maximum temperature and precipitation of the previous November, and by minimum temperature in July and precipitation in October. It is known that turgor, which is closely linked with water status in a tree (Steppe et al., 2015), has not only an impact on cell expansion, but also on biosynthesis of secondary cell wall (Proseus and Boyer, 2006). On the other hand, anatomical variations in xylem depend on rate and duration of differentiation processes (Skene, 1972), which determine the amount and properties of wood (Hölttä et al., 2010). Thus, both water and carbon affect seasonal radial growth resulted in wood anatomy, which influence xylem hydraulic conductivity and cavitation vulnerability (Steppe et al., 2015).

The thickest cell walls in earlywood and latewood were found in spruce at the lower site PA, with the widest tree rings. According to Hacke and Sperry (2001), vulnerability to cavitation depends on the mechanical strength of the conduits, so tracheids in drier environments usually have thicker cell walls. However, Gindl et al. (2001) measured the lignin content in tracheids of Norway spruce from two altitudes; 580 and 1260 m, and observed that the low-elevation trees had wider growth rings, with thicker cell walls. In contrast, the lignin content was negatively correlated to the cell wall thickness. The authors hypothesized that trees growing at higher altitudes compensate for the thinner cell walls with an increased lignin content, which helps to maintain the mechanical integrity of the xylem (Gindl et al., 2001).

Even relatively small differences in the xylem structure of spruce from three temperate regions can have a considerable influence on the annual hydraulic properties of the tree ring (Tyree and Zimmermann, 2010). Namely, small increases in radial conduit diameter result in large changes in conductivity due to the direct relationship between flow and radius to the fourth power (Tyree and Zimmermann, 2010). Moreover, other anatomical characteristics of tracheids, such as their length (Mäkinen et al., 2008) and pit structure (Hacke and Jansen, 2009) may have further contributed to regulation of the hydraulic trade-off. The differences could be an adaptation of trees to function optimally in local environmental conditions (Bryukhanova and Fonti, 2013).

### Phloem cell characteristics

As found for the widths of the annual phloem increment (Prislan et al., 2013; Gričar et al., 2014b), the dimensions of the sieve elements also seem to be predominantly site-specific characteristics in Norway spruce. In general, phloem anatomical parameters varied among the sites but were fairly uniform among years. The number of late phloem cells as well as their radial dimension were mainly affected by precipitation in late autumn and maximum temperature in winter. The number of early phloem cells and dimension of all phloem cells were also positively influenced by precipitation in April. In addition, radial dimension of initial early phloem sieve cells was determined by maximum temperature or minimum in the period January–March. Favorable conditions in late autumn and winter positively affect phloem growth and carbon reserves in the following year (Lebourgeois et al., 2010).

Similarly as in wood, late phloem was also more variable than early phloem. These findings confirm again our previous assumptions that stable phloem formation patterns and its structure, as previously repeatedly reported, is found only in trees of similar age, position in stand, vigor and vitality, which are growing in similar environments (Gričar et al., 2014a). As in xylem, the conductive capacity of phloem cells is not only affected by their anatomical structure, such as conduit size and number, but also by sieve pore size and frequency along the pathway (Mullendore et al., 2010). Jyske and Hölttä (2015) reported that phloem conduits were slightly narrower than xylem conduits. In our case, that was similar only for the early part of the annual rings, whereas in the increments formed in the second part of the growing season, it was just the opposite. Thus, tracheids in earlywood were on average 12–30% wider and in latewood 30–50% narrower than sieve cells in early and late phloem, respectively. These ratios were different among the sites but uniform among the years. There was no linkage between the radial lumen size of initial and terminal cells in xylem and phloem cells at an individual location. Thus, the variation in the structure of annual xylem and phloem increments in Norway spruce clearly shows that plasticity in the seasonal dynamics of cambial cell production and cell differentiation exists on both xylem and phloem sides. Xylem has different requirements for mechanical and hydraulic safety (Sperry et al., 2008), reflecting in larger functional areas and more rigid cell walls than in phloem. This results in lower conduit frequency per unit of conducting area but in higher mechanical support (Jyske and Hölttä, 2015). In xylem, sap transport usually occurs over several sapwood rings, with declining conductivity toward the inner sapwood (Spicer and Gartner, 2001), whereas phloem sieve elements function for only one to two growing seasons. Tree survival therefore depends on the yearly formation of new phloem to maintain and extend translocation pathways for photosynthates and biomolecules (Evert, 2006). However, the relevance of the annual hydraulic plasticity of the xylem tissue on future performance needs to be adjusted to its relative contribution to the whole sapwood and not only to the outermost annual increment (Jyske and Hölttä, 2015). In addition to sieve cells (and Strassburger cells), ray and axial parenchyma networks constitute annual phloem increments, which potentially serve as an important carbohydrate and water reserve (Rosell et al., 2014). Since the variability of late phloem is higher than that of early phloem, a variation in phloem annual increments can greatly affect the amount of axial parenchyma in the phloem. No such information exists to date. However, it deserves more in depth investigation in order to understand better the role of parenchyma cells on the whole functioning of trees in different environments.

### Climate-growth relationship

Analysis of the climatic impact on the radial increment of Norway spruce from the three studied locations revealed site-specific growth sensitivities to local climatic conditions. Radial growth of spruce was on all three sites only partly dependant on climatic factors, including maximum and minimum temperature and precipitation. Tree-ring widths of spruce growing in temperate forests under average climatic conditions are known to respond less intensely to climatic variation than trees growing in extreme conditions (Mäkinen et al., 2003). Local site conditions, tree age, tree competition and tree vitality, as well as different forest management practices can be more important drivers of local-scale growth trends than the regional climate signal (Levanič et al., 2009).

The most important climatic parameters affecting the radial growth of spruce at the Slovenian lower site PA was found to be a positive effect of a warm January and negative affect of a hot summer (June–July). Spruce at the Alpine site ME showed a significant positive response to March precipitation, while spruce at the Czech site RN mainly negatively responded to a warm previous September and positively to a warm late winter (February–March). Other research groups have reported that the most important climatic parameters affecting spruce growth throughout Europe are summer temperatures, and the summer and autumn temperatures of the previous year, especially July and September (e.g., Savva et al., 2006; Bošel'a et al., 2014). However, the response to the same climatic driver can work in opposite directions at different sites. For spruce from higher elevations, the response to summer temperature is generally positive, since temperature is a limiting factor for growth, whereas at lower sites, the effect of summer temperature is negative, because it is usually related to a decrease in precipitation, a limiting factor for low-altitude spruce (e.g., Levanič et al., 2009; Lebourgeois et al., 2010). In our study, interestingly, growth was more weakly correlated with precipitation than with temperature. In addition, precipitation in spring seemed to be mainly important for the Alpine site ME. This could be explained by the fact that none of our sites is precipitation limited. Even at the Czech site RN with annual precipitation of only 600 mm, rain falls predominantly during the growing period.

Earlywood widths showed a similar relation to climatic conditions as tree-ring widths. Nevertheless, earlywood width was generally less dependent on climatic conditions than that of latewood. Some authors have ascribed the low dependence of earlywood width on climate to more endogenous control of its formation (Bošel'a et al., 2014). The latewood width at all three sites was negatively affected by a hot summer and also to the climatic conditions of the previous autumn. In terms of the latewood width, trees at the Alpine site ME showed the highest growth dependence on climatic conditions in July, whereby wet and less hot conditions positively affected the amount of latewood. This is probably mainly related to prolongation of wood (latewood) formation in this period. Our findings are in contrast with previous observations (e.g., Levanič et al., 2009) but, as already mentioned, the sites selected for this study were sufficiently supplied with precipitation. In addition, microtopography, soil properties and rock type, which differ among the selected sites, influence runoff, distribution and water movement in the soil. Acid granodiorite rock type at RN, for example, is able to retain more water than dolomite at PA or limestone at ME.

Other studies have also highlighted a highly negative response of tree-ring width to the summer temperature of the previous year (e.g., Andreassen et al., 2006; Bouriaud and Popa, 2009). The authors explained this relationship by the stimulation of cone production after a warm summer for trees that have reached maturity (Andreassen et al., 2006) or high respiration rates triggered by high temperatures, which would decrease the carbohydrate reserves available for needle development and growth initiation during early phases of the growing season (Bouriaud and Popa, 2009).

## Conclusions

In temperate regions, where climatic conditions are favorable for growth of Norway spruce, variation in the structure of xylem and phloem increments is mainly observed at the site level rather than on a temporal scale. In addition, xylem and phloem tissues formed in the first part of the growing season seem to be more stable in structure, which indicates their priority over latewood wood and late phloem for tree performance. Analysis of the long-term climatic impact on the radial increment of Norway spruce from the three studied locations confirmed site-specific growth sensitivities to local climatic conditions. Earlywood widths showed a similar relation to climatic conditions as tree-ring widths, although earlywood width was generally less dependent on climatic conditions than that of latewood. Radial growth was in general less dependent on precipitation than on temperature, which could be explained by the fact that none of the study sites is precipitation limited. Radial growth of spruce was on all three sites only partly dependant on climatic factors, which confirms the strategy of spruce to adapt the structure of wood and phloem increments to function optimally in local conditions.

Ability of plants to adapt to the surrounding environmental conditions is important since climate is always changing, both in terms of long-term average values and in terms of frequency and severity of extreme events. Adaptation is thus characteristic of xylem and phloem cell parameters and increment widths. Knowledge of intra-annual radial growth could provide valuable information on adaptation strategies of spruce to local environmental conditions and will help to improve interpretation of the relevance of such high annual plasticity of secondary tissues on future tree growth.

## Author contributions

JG—developed the concept of the paper, wrote the paper, and together with KČ performed wood-anatomical analysis for the Slovenian sites; PP, ML—carried out the statistical analysis, wrote the statistical parts of the paper and prepared the figures; VG, JH, HV—performed wood-anatomical analysis for the Czech site. KČ was responsible for the Slovenian sampling, section preparation and climate data. All authors discussed and commented on the manuscript.

## Funding

This work was supported by the Slovenian Research Agency, young researchers' program and programs P4-0015 and P4-0107, by the Spanish Science and Innovation Ministry (MICINN), the ELENA program (CGL2012-31668), by the FEDER program of the European Union and by the European Social Fund and the state budget of the Czech Republic, Project Indicators of Trees Vitality Reg. No. CZ.1.07/2.3.00/20.0265. The cooperation among the international partners was supported by COST Action FP1106, STReESS.

### Conflict of interest statement

The authors declare that the research was conducted in the absence of any commercial or financial relationships that could be construed as a potential conflict of interest.
